# Early Cenozoic Differentiation of Polar Marine Faunas

**DOI:** 10.1371/journal.pone.0054139

**Published:** 2013-01-16

**Authors:** J. Alistair Crame

**Affiliations:** British Antarctic Survey, Natural Environment Research Council, Cambridge, United Kingdom; Technical University of Denmark, Denmark

## Abstract

The widespread assumption that the origin of polar marine faunas is linked to the onset of major global cooling in the Late Eocene – Early Oligocene is being increasingly challenged. The Antarctic fossil record in particular is suggesting that some modern Southern Ocean taxa may have Early Eocene or even Paleocene origins, i.e. well within the Early Cenozoic greenhouse world. A global analysis of one of the largest marine clades at the present day, the Neogastropoda, indicates that not only is there a decrease in the number of species from the tropics to the poles but also a decrease in the evenness of their distribution. A small number of neogastropod families with predominantly generalist trophic strategies at both poles points to the key role of seasonality in structuring the highest latitude marine assemblages. A distinct latitudinal gradient in seasonality is temperature-invariant and would have operated through periods of global warmth such as the Early Cenozoic. To test this concept a second global analysis was undertaken of earliest Cenozoic (Paleocene) neogastropods and this does indeed show a certain degree of faunal differentiation at both poles. The Buccinidae, s.l. is especially well developed at this time, and this is a major generalist taxon at the present day. There is an element of asymmetry associated with this development of Paleocene polar faunas in that those in the south are more strongly differentiated than their northern counterparts; this can in turn be linked to the already substantial isolation of the southern high latitudes. The key role of seasonality in the formation of polar marine faunas has implications for contemporary ecosystem structure and stability.

## Introduction

It would seem only logical to place the origin of modern polar marine faunas largely within the major global cooling event that occurred between the late Middle Eocene and the Eocene – Oligocene boundary (i.e. approximately 34–41 m.y. ago) [1], [2]. A variety of both paleontological and phylogenetic evidence has been presented over the years to suggest that this is the case in both the Antarctic and North Pacific, with the latter subsequently providing the bulk of the modern Arctic fauna [3]–[5]. Important confirmation that such a process did indeed take place in Antarctica has been obtained recently from the highest stratigraphic levels of the Eocene La Meseta Formation, Seymour Island, Antarctic Peninsula. Here, the sudden appearance of cold-water assemblages of both marine invertebrates and vertebrates has been dated to almost exactly this time interval [6]–[8]. It has been estimated that the invertebrates suffered a 50% drop in taxonomic diversity at this time [9] and this may well have been a period of significant steepening of latitudinal gradients in taxonomic diversity worldwide [10], [11].

Nevertheless, there has also been a small but persistent volume of evidence to suggest that Antarctic marine invertebrate faunas in particular may be of considerably greater antiquity. This again comes from both paleontological and phylogenetic sources and is such as to suggest that some modern taxa are of Early Cenozoic, Cretaceous, or even greater age [12]–[18]. In a recent comprehensive taxonomic reassessment of Paleogene molluscan faunas from Antarctica, Beu [19] showed that more than 15% of the Paleocene taxa and 30% of the Early – Middle Eocene could be referred to modern genera. Furthermore, when both the gastropod and bivalve components are considered at the family level, remarkably persistent compositional trends can be traced through much of the Cenozoic. This is particularly so within the gastropods where families/family groups such as Trochidae, Naticidae, Conoidea ( =  Turridae, s.l.) and above all the Buccinidae, s.l. maintain their dominance from the Early Paleocene through to the present day. The modern Antarctic molluscan fauna, at least, may have had its roots very firmly within the Early Cenozoic greenhouse world [19], [20].

It is therefore a matter of considerable interest and importance to establish the nature and scale of polar marine faunal differentiation through the Early Cenozoic greenhouse interval. If certain elements of modern faunas were indeed flourishing at that time then it would indicate that factors other than low temperature per se played a key role in their formation. It is the intention of this study to further this line of enquiry using a combination of datasets from both the modern and fossil records to isolate the key ecological parameters affecting the formation of polar marine faunas.

## Methods

Even after taking into account the inherent biases within the fossil record, it is apparent that shelled gastropods underwent a dramatic evolutionary radiation globally through the Cenozoic era [21]–[24]. From comparatively low numbers immediately following the K – Pg mass extinction event they rose to some 60,000+ species at the present day [25]–[27] and, with the possible exception of the polychaetes and nematodes (whose total numbers of species are still very poorly known), are the most taxonomically diverse group in modern shallow seas. By far the largest gastropod clade at the present day is the Neogastropoda which probably contains in the region of 26,000 species (Appendix S1). As the name implies, it is also the youngest clade, with a time of origin in the Early Cretaceous and major phase (or phases) of radiation throughout the Cenozoic [28]–[30]. Geographically widespread, it is ideal for regional scale biogeographical analyses and in this study a direct comparison will be made between the living neogastropod faunas of both polar regions and a composite tropical fauna to see the end-product of clade differentiation through the Cenozoic. This will then be compared directly with approximately similar datasets taken from the Paleocene fossil record. Can regional patterns of faunal differentiation seen at the present day be detected as far back as the Paleocene (i.e. the initial epoch of the Cenozoic era)?

The modern tropical neogastropod fauna used in this study is an average of that found at six principal localities: two from the Americas, Tropical Western Atlantic and Panamic province, and four from the western Pacific: Philippines, Guam, New Caledonia and French Polynesia (with further details of all these localities being given in the Appendix S1). The Arctic fauna comprises a compilation of all taxa occurring north of 60°N, but with the Bering Sea and Sea of Okhotsk excluded. The Antarctic fauna includes all taxa currently recorded from south of the Polar Front, and is a mixture of both shelf and bathyal taxa (which intergrade in the Antarctic) (Appendix S1). A comparison of continental shelf areas shows either of the two polar regions to be very much larger than the six tropical localities combined (Appendix S1, table 1).

Paleocene gastropod data were selected for 19 regional localities ranging from 63°N to 64°S paleolatitude; these were obtained from a variety of published sources, supplemented by the Paleobiology Database (http://paleodb.org), and, in a small number of cases, reference collections (Appendix S1). Key selection criteria included a clear demonstration that the fauna was reasonably taxonomically complete, and restricted, in essence, to a single lithostratigraphic formation. It will become apparent from the Appendix S1 that these 19 faunas vary somewhat in age and thus were not strictly contemporaneous. Nevertheless, given the relatively imprecise nature of Paleocene dating on a global scale, and the apparent success of using time-averaged faunas in similar Mesozoic biogeographical investigations [31]–[33], this was not thought to be a major impediment to the study.

In the following analysis the highest-latitude Paleocene gastropod fauna from the Northern Hemisphere, the prolific assemblage from West Greenland (64°N) is counterbalanced by a composite southern high latitude fauna comprising assemblages from southernmost Patagonia, Antarctic Peninsula, S.E. Australia and New Zealand (55°–64°S) (Appendix S1). Each of the latter faunas contains elements of Zinsmeister’s [34] distinctive Weddellian Province and there is a considerable degree of faunal overlap between them [35], [36]. Although a low-latitude gastropod fauna can be traced from S.W. Nigeria (2°S) through the Western Desert of Egypt (14°N) to S.E. Pakistan (5°S) [37], [38] (Appendix S1), it would appear to be significantly less diverse than that present in N.W. Europe. Coral – algal patch and larger reef structures were relatively common in western Tethys during the Early Paleocene and these clearly extended westwards into the Paris and Belgian basins (43°–44°N) [39]–[41]. As the Danian gastropod faunas from both these regions show strong similarities with both N.W. Germany (45°N) [42], [43] and Fakse, Denmark (49°N) (Appendix S1), all four localities have been combined into a Paleocene “Tropics – N.W. Europe” category. Faunas from six separate Paleocene formations on the U.S. Gulf Coast (35°–39°N) have not been combined as the precise lateral equivalence of stratigraphic levels in the western gulf (i.e. Texas) and eastern gulf (i.e. Alabama) has yet to be fully established [44].

Quantitative comparisons between tropical and polar/subpolar faunas were made for both the present day and Paleocene using a series of standard statistical tests and an analysis of rank/abundance distributions. The family/family group level is used in these analyses and particular attention paid in the ensuing discussion to their trophic characteristics.

## Results

### a) Distribution of Modern Neogastropods

**Figure 1:**
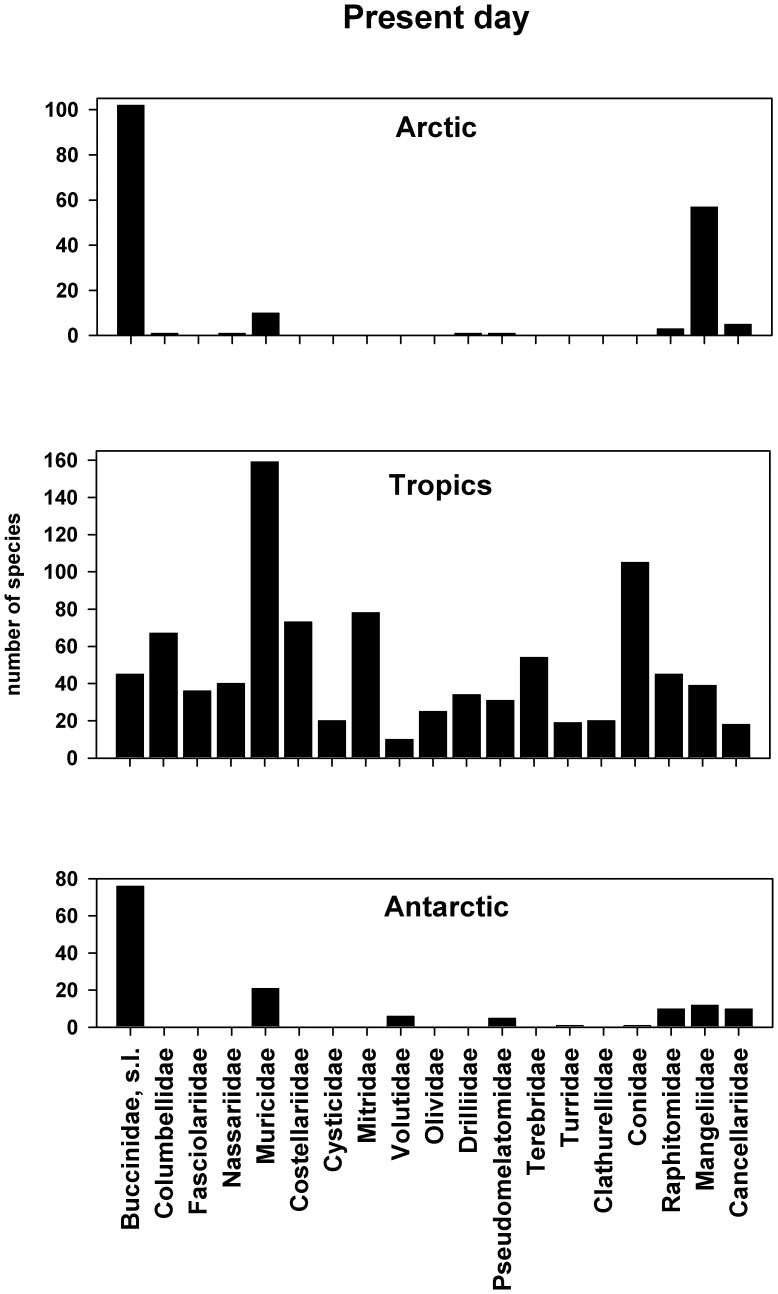
Comparison of present day regional neogastropod faunas between the Arctic, Tropics and Antarctic. The histograms depict the number of species occurring within 18 common neogastropod families and one family group (Buccinidae, s.l.) at each locality. Further details of how these three faunas were compiled are given in both the text and Appendix S1.

It should be emphasised that the total number of gastropod species occurring at the present day in the Indonesian – Philippines core region of the Indo-West Pacific province is currently unknown but could be at least 10,000 species [45]. The steepest regional latitudinal gradients in gastropod diversity occur from both this region and the core of the Atlantic – Caribbean – East Pacific province (*sensu* [46], with the estimated number of species being in excess of 5,000 species – JAC unpublished data) to both poles (Arctic –388 species, Antarctic –450 species). To get a conservative estimate of tropical neogastropod diversity at the present day, a mean value was taken from the six selected localities within the 19 commonest families; these were then compared directly with absolute values for both polar regions (Fig. 1). When such a comparison is made it is apparent that there are more than twice as many families per clade in the tropics (n = 19.00) as at the poles (Arctic = 9.00, Antarctic = 9.00; Appendix S1, table 2) (with these differences being statistically significant using a Wilcoxon Signed Rank Test, P<0.05), and considerably more species per family (Tropics = 48.32, Arctic = 20.11, Antarctic = 16.22) (significant in both cases at P<0.05). However, it is clearly not a case of there simply being fewer species in each of these 19 families at the poles, as in both cases the Buccinidae, s.l. is clearly the dominant family. Together with the Mangeliidae it comprises 89% (by species number) of all Arctic neogastropods, and in the Antarctic the only other significant occurrences include a comparatively small number of Muricidae and former members of the Turridae, s.l., now reclassified within the Conoidea families Pseudomelatomidae, Raphitomidae and Mangeliidae (Buccinoidea+Conoidea = 74% of all Antarctic neogastropods). Although the distribution patterns for both the Arctic and Antarctic are highly significantly different from that of the Tropics (Kolmogorov-Smirnov Two-Sample Test, P<0.001), they are not significantly different from each other (P>0.05).

**Figure 2:**
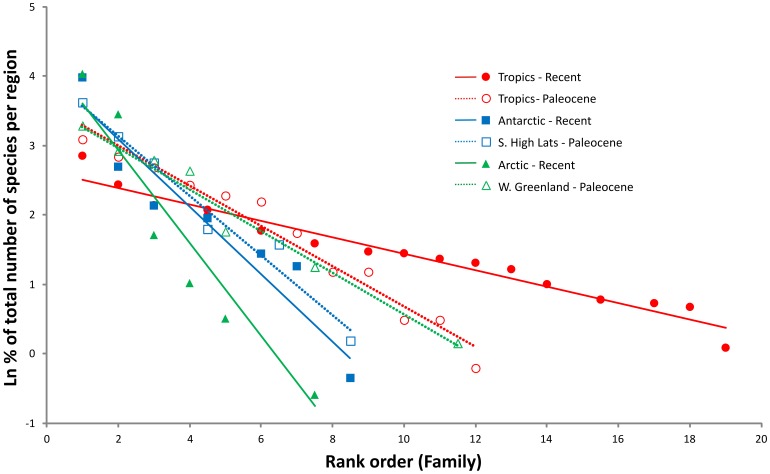
Rank/abundance plots for three Recent and three Paleocene regional neogastropod faunas. Lines shown are fitted linear regressions for each of the six faunas. Further details of how the plots were constructed, and the regression lines compared, are given in the text.

To investigate these patterns further, rank/abundance distributions were calculated following a procedure advocated by Magurran [47]. This involves plotting rank order of the families in each fauna (i.e. from most to least speciose) against log% of the total number of species per fauna (Fig. 2). Classical linear regressions were fitted to the three distributions obtained and both the slopes and Y intercepts of these compared using an ANCOVA procedure in Minitab 15. These three lines were compared with each other and also with three generated for corresponding Paleocene faunas (see below) using Bonferroni Simultaneous Tests. This analysis confirms that both the modern Arctic and Antarctic faunas have much steeper slopes and are thus much less evenly distributed than that of the Tropics (P = 0.000 and P = 0.0165, respectively) (Fig. 2). Again, they are both highly significantly different from the Tropics, but not significantly different from each other (P = 0.4392).

### b) Distribution of Paleocene Neogastropods

**Figure 3:**
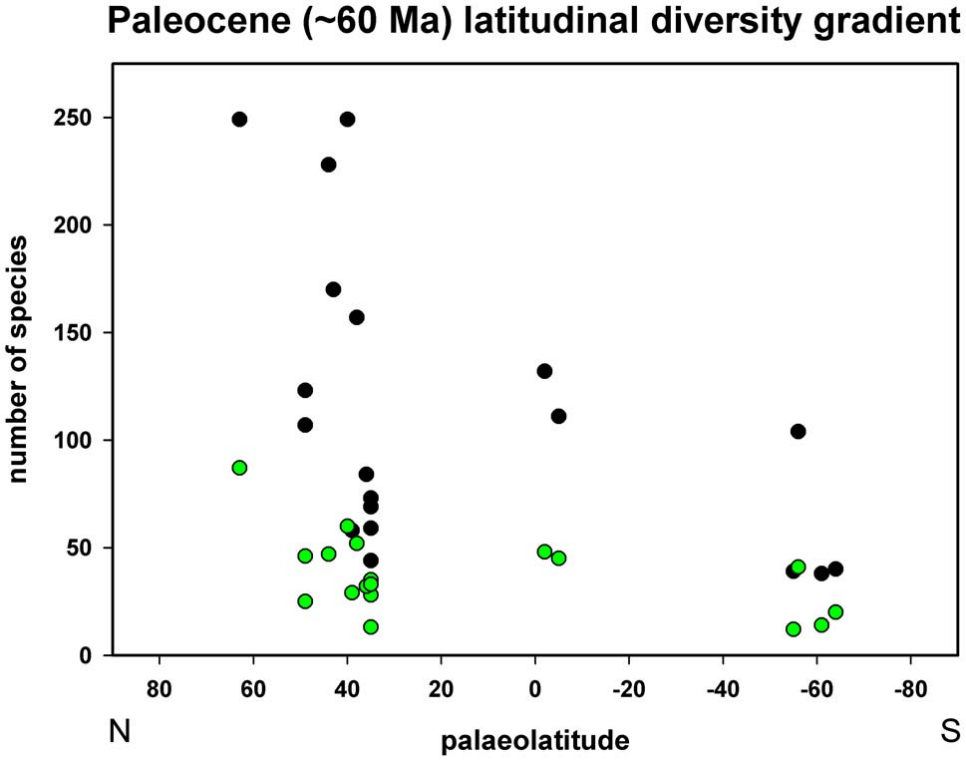
Paleocene (∼60 Ma) latitudinal diversity gradient. Full details of how the 19 Paleocene faunas on which this gradient is based were compiled are given in Appendix S1. Latitudinal gradient for neogastropods only shown in green. Paleolatitudes taken from the Paleobiology Database.

At first sight the distribution pattern obtained when the total number of gastropod species from each of the 19 regional localities is plotted against paleolatitude seems to be anomalous (Fig. 3). Maximum numbers of species, and in particular those from the West Greenland, southern Poland and Belgium localities (Appendix S1), occur in the interval 40°–63°N rather than a more equatorial position and this could perhaps be taken as an indication of a very imperfect fossil record. Nevertheless, it has to be borne in mind that the tropics extended to considerably higher paleolatitudes in the Paleocene and a coral reef belt can be traced through at least part of N.W. Europe (see above). Both at the present day and in the past there is a strong correlation between the taxonomic diversity of reef-building and reef-dwelling organisms such as gastropods [48]–[50]. Although corals occur extensively in the more equatorial belt of limestones traced from S.W. Nigeria, through Egypt, to S.E. Pakistan, reefs have not been recorded in this region. In addition, Paleocene reefs are unknown in the mid- to high-latitudes of the Southern Hemisphere [39]. It is possible that the high diversity value for West Greenland represents, at least in part, a northward extension of the N.W. European tropical fauna by some form of warm-water current. This could be analogous to the northward extension of tropical/subtropical faunas at the present day in the western Pacific by the Kuroshio current [51]. In any event it is apparent that there must have been a very steep drop in taxonomic diversity at approximately 50°–60°N, similar in many ways to that seen at the edge of the modern coral reef belt at 20°–30°N [51], [52]. Contrary to recent reports from the Early Cenozoic terrestrial realm [53]–[55], there could in fact have been a very steep latitudinal diversity gradient in the Early Cenozoic marine realm at a high paleolatitude (Fig. 3).

It is important to emphasise that a distinct Early Paleocene (Danian) Arctic Ocean marine fauna can be detected to the north of the West Greenland locality (i.e. at 70°+N). Even though the Arctic Ocean was very much smaller at this time and probably only had tenuous connections with the rest of the world ocean, elements of this fauna can be traced from Ocean Point, Alaska (upper Prince Creek Formation), through Ellesmere Island (Mt. Moore Formation) to Svalbard (Barentsburg and Grumentdalen formations) [56]–[62]. So far only approximately 11 gastropod species (including just one neogastropod) have been indentified within this fauna but both they and the more common bivalves have clear temperate affinities. This Arctic Ocean marine fauna also has strong taxonomic links with the similarly-aged Cannonball Formation of North and South Dakota, a unit that has been widely interpreted as being the product of a major southerly incursion of north polar waters [62], [63]. Although the gastropod fauna of the Cannonball Formation is in need of taxonomic revision, it is well preserved and known to comprise at least 29 species with strong temperate affinities [64]. If this was taken to be representative of a true Arctic Ocean locality, then it would add further weight to the concept of a very steep Paleocene latitudinal diversity gradient in the highest northern latitudes (Fig. 3). Taxonomic links between the Cannonball Formation and Agatdal Formation of West Greenland [65], [66] (Appendix S1) suggest that the latter fauna is indeed a genuine admixture of cold- and warm-water types (see below).

As might be expected, the Neogastropoda forms a smaller proportion of the global Paleocene gastropod fauna (34%) than at the present day (42%), but these differences are not statistically significant (χ^2^ test, P>0.05). It is still the largest clade but when viewed on its own has a much flatter latitudinal profile than the total gastropod fauna (Fig. 3). It is possible that the only steep latitudinal gradient in Paleocene neogastropods would have been from West Greenland northwards into the Arctic Ocean. In the Paleocene a significant proportion of tropical gastropod faunas was still composed of clades such as the Vetigastropoda and, in particular, the Cerithioidea.

**Figure 4:**
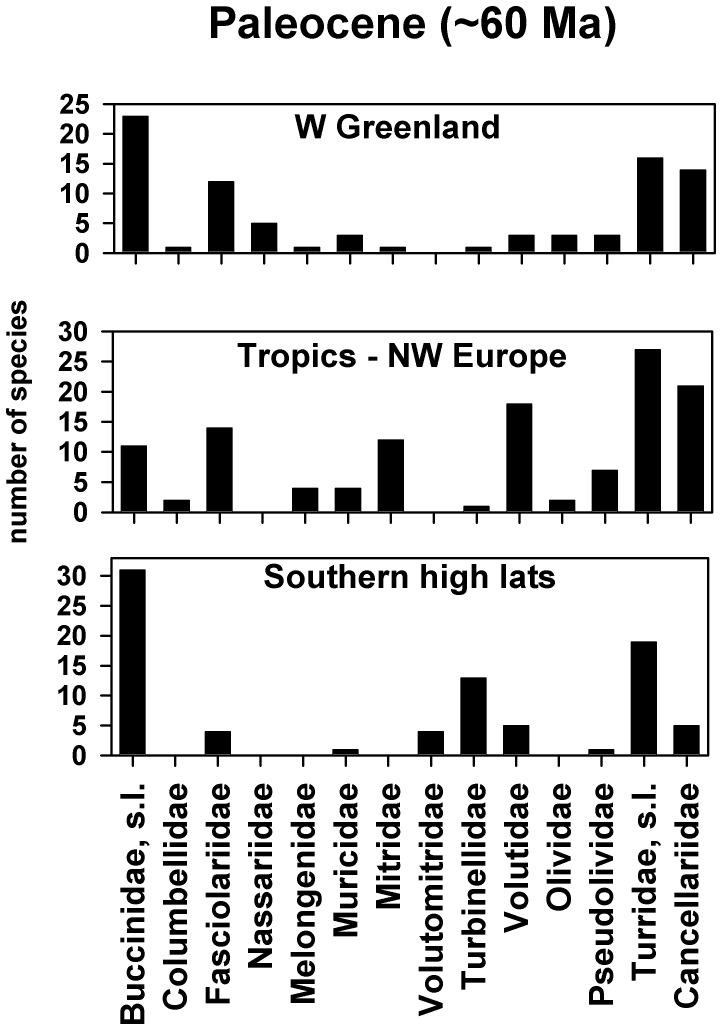
Comparison of Paleocene (∼60 Ma) regional neogastropod faunas between West Greenland, Tropics – N.W. Europe, and Southern high latitudes localities. The histograms depict the number of species occurring within 12 common neogastropod families and two family groups (Buccinidae, s.l. and Turridae, s.l.). Further details of how these three faunas were compiled are given in both the text and Appendix S1.

It is apparent that the contrast in distribution of neogastropods between the three regional faunas is not so strong in the Paleocene as at the present day (Fig. 4). The Southern high latitudes is the most distinctive fauna, where there is again a strong domination by the Buccinidae, s.l. taxon. In the Antarctic this category includes a probable representative of the Southern Ocean genus *Probuccinum*, as well as a distinctive buccinid that is close to the modern Arctic genus, *Colus* [67]. Similarly, the Paleocene of SE Australia and New Zealand has yielded *Cominella*, *Austrofusus*, *Buccinulum* and *Penion*, all of which are known from Australasian regions at the present day [35]. The Turridae, s.l. (see Appendix S1 for notes on the use of this taxon in the Paleocene) is the second most prominent family/family group in the Southern high latitudes and includes at least ten distinct genera from four modern conoidean families. However, no modern Southern Ocean genera have yet been recognised in this fauna. The only other prominent family in this region is the Turbinellidae (Fig. 4), but nearly all of these occurrences are from just one locality, New Zealand.

When the Paleocene rank/abundance plots are considered (Fig. 2) it is interesting to note that the fitted regression line for the Southern high latitudes plots close to that for the modern Antarctic, from which it does not differ statistically (P = 1). However, it also has to be pointed out that, although this line plots away from those of the other two Paleocene localities, it cannot be statistically separated from them either. In addition there is no significant difference between all three distributions (Fig. 4) using a K-S two sample test. Nevertheless, there are some reasonably strong resemblances between the two polar localities in that Buccinidae, s.l. and Turridae, s.l. are again the two numerically dominant family groups in the West Greenland fauna (Fig. 4). Unfortunately, virtually all of the buccinid determinations in the study by Kollmann and Peel [66] can only be regarded as provisional and it is not possible to say with any degree of certainty whether there are representatives of modern genera in this fauna. This is particularly so of identifications of southern genera such as *Penion* and *Cominella*, which seem most unlikely (A.G. Beu, pers. comm. 2012). In addition, although the Turridae, s.l. fauna from West Greenland contains representatives of approximately five modern conoidean families, there appear to be no Mangeliidae (the dominant Arctic family at the present day) [66]. Two other prominent families in the West Greenland fauna are the Fasciolariidae and Cancellariidae (Fig. 4).

Although a detailed comparison between West Greenland and Tropics – N.W. Europe must await further taxonomic investigations, it is almost certain that at least a small number of species are common to the two regions. The tropical nature of the West Greenland fauna is reinforced by various representatives from families such as Neritidae, Cypraeidae and Harpidae, but counterbalanced against this are a number of significant differences between the two regions. The relative proportions of both the Vetigastropoda and Cerithioidea clades are much smaller in this fauna, as are the numbers of neogastropods in families such as the Mitridae and Volutidae (Fig. 4). The Buccinidae, s.l. is clearly much more diverse and there are at least four taxa from the hypsogastropod family Aporrhaidae, which is extremely rare in the tropical localities used in this study. The logical conclusion would seem to be that this is a genuinely mixed fauna comprising both tropical and temperate elements.

The rank/abundance plots reveal the West Greenland and Tropics – N.W. Europe faunas to be almost identical in both slope and intercept (Fig. 2). The former of these is highly significantly different from the modern Arctic fauna (P = 0.0158) but the latter, although plotting away from the modern tropics is not significantly different from it (P = 0.4457). The difference between West Greenland and its modern counterpart is clearly very much greater than that between the Southern high latitudes and present day Antarctica.

## Discussion

Although there is still a considerable degree of detail to be filled in, especially from the mid-latitudes, it is likely that regional latitudinal gradients in taxonomic diversity exhibited by modern gastropods from the tropics to the poles are the steepest in the entire marine realm. The fact that this pattern is substantially repeated in the largest component clade, the Neogastropoda (Fig. 1), is particularly interesting as it must be attributable largely to evolutionary processes occurring through the Cenozoic era (i.e. the last 65 m.y.). The balance of evidence would perhaps suggest that over this period of time the tropics have acted as the primary source of new taxa which have then gradually disseminated into higher latitudes; in such a scenario the tropics can be regarded as an evolutionary source, and the poles as evolutionary sinks [68], [69]. In essence, regional latitudinal diversity gradients are the product of a large-scale diffusion process from the tropics to the poles.

But examination of the distribution patterns displayed in Figure 1 suggests that the end product of 65 m.y. of evolution is something more than a random accumulation of neogastropod taxa in the polar regions. In both cases there has been a concentration of species within three main taxonomic categories: Buccinidae, s.l., Muricidae, and closely related conoidean families such as Mangeliidae, Raphitomidae and Pseudomelatomidae. Of course, it should be emphasised that at lower taxonomic levels the Buccinidae, s.l. in the Arctic can be divided into five subfamilies, Colinae (55%), Buccininae (27%), Volutopsinae (9%), Beringiinae (7%) and Ancistrolepisinae (2%), none of which has been identified with certainty in the Antarctic [3], [70], [71]. It is possible that all of the distinctive Southern Ocean buccinids, including genera such as *Chlanidota*, *Pareuthria*, *Probuccinum* and *Prosipho*, could be included in the subfamily (or tribe?) Buccinulinae [27], [72] but such an assignment still needs to be fully substantiated [73], [74]. *Lirabuccinum*, a northern cool-water buccinid, does show a number of strong similarities with southern temperate forms such as *Buccinulum* [72], [75] but there are no true bipolar genera between the Arctic and Antarctic.

Members of the Buccinidae, s.l. are generalist carnivores employing both predatory and scavenging modes of feeding. Their prey is known to include bivalves, polychaetes, small crustaceans, cirripedes, eggs and carrion, and there is evidence to show that polar taxa have a much wider range of diets than their tropical counterparts [76], [77]. In comparison, members of the Mangeliidae, Raphitomidae and Pseudomelatomidae are thought to feed very largely on polychaetes, but these are in turn deposit-feeders and form a very stable food resource in an otherwise strongly seasonal environment [77]. All three of these families are indeed more common in the tropics (Fig. 1) but it is the overall ratio of generalist to specialist feeding types that is very much higher in polar than tropical neogastropods. If we take Buccinidae, s.l. plus Conoidea (except Conidae and Turridae) as a measure of generalist feeders within a regional fauna then the 89% they comprise in the Arctic and 74% in the Antarctic can be compared with a figure of just 32% for the Tropics (with these polar – tropical comparisons being highly significantly different; χ^2^ test, P = 0.000). The diets of polar muricids are still poorly known but there is evidence to show that at least one common Antarctic species, *Trophon longstaffi*, feeds only very infrequently and on a variety of bivalve and brachiopod prey. It is characterized by extremely low metabolic rates and overall would seem to be very well adapted to long periods of limited food availability [78].

Thus the comparatively small number of successful neogastropod families and family groups in the high-latitude and polar regions show the characteristics of ecological generalists, and it is likely that this phenomenon is exhibited in other taxonomic groups too. For example, within the benthic foraminiferans there is a distinctive polar *Epistominella exigua – Alabaminella weddellensis* assemblage that comprises a group of opportunistic phytodetritovores [79], [80], and similar patterns of differentiation may be shown by the protobranch bivalves, as well as certain groups of isopods and cumaceans [81], [82]. But even if a regime of strongly seasonal primary productivity does favour the development of more generalist clades in the polar regions, it does not necessarily explain why taxonomic diversity as a whole should be so low. It has been argued that variable food supply must have had an effect on both population density and population growth as resource exploitation is limited to only part of the annual cycle of production [83]. Such an effect may have been particularly severe in predominantly predatory groups such as the neogastropods where the ability to specialize in diet would have been much more limited than in the tropics. However, logical as these ideas may seem, they have not yet been fully tested in a rigorous manner.

We may take as a valuable working hypothesis that the latitudinal gradient in the seasonality of primary productivity may be of prime importance in determining the structure and composition of polar marine faunas [79], [84]. Such a gradient is, of course, temperature – independent and could equally well apply in a greenhouse as an icehouse world. There is some evidence to suggest that both the origination and extinction rates of polar generalists are comparatively low, and that they comprise relatively stable assemblages over long periods of time [85], [86]. Only a relatively small number of such taxa become established in the polar regions, but they then tend to be temporally persistent.

With only 19 regional localities available for analysis it is not possible to be certain about the overall form of latitudinal diversity gradients in Paleocene gastropods (Fig. 3). There is some evidence from the Northern Hemisphere to suggest that there was a very steep drop in taxonomic diversity values at 40°–60°N and this could reflect the edge of a tropical reef belt. Unfortunately, there are insufficient data points to indicate whether there is a matching drop-off in values in the southern mid- to high-latitudes, but there is at least some evidence from the terrestrial realm to indicate that the tropics reached to 40°–50°S in southern South America [87], [88]. It might well be that the Early Cenozoic tropics were characterised by a broad plateau of relatively high diversity values stretching from approximately 50°N to 50°S and then flanked by steep gradients to both poles; however, such a concept has yet to be fully substantiated. It is more certain that, even though neogastropod latitudinal gradients were much shallower than their counterparts at the present day, they show clear indications of differentiation into polar faunas that exhibit the early stages of dominance by a small number of families/family groups, and a tropical fauna with a more even distribution of taxa. Buccinidae, s.l. are particularly prominent in both polar faunas and would seem to have been the product of a distinct earliest Cenozoic radiation event [66], [67], [89].

There is some evidence to suggest that there may have been a considerable degree of asymmetry in the development of the two polar neogastropod faunas. Whereas the Paleocene Southern high latitudes fauna sits close to its modern counterpart in the rank/abundance plots, West Greenland does not (Fig. 2). The Southern high latitudes fauna contains representatives of several living genera and looks altogether more modern in aspect than the corresponding fauna for West Greenland. Such a disparity may perhaps reflect nothing more than degree of physical separation of the respective ocean basins, for although not yet totally isolated there was already a sizeable Southern Ocean south of 60° paleolatitude in the earliest Cenozoic. The Arctic Ocean basin, in comparison, was very much smaller and certainly not connected to the extensive North Pacific at the time. It would appear that there are some Paleocene marine strata in the north-west Pacific region but the earliest records of gastropod genera that dominate cold-water molluscan assemblages of the North Pacific at the present day are from the Middle Eocene [90], [91].

Clearly there were many intermediate stages in the evolution of the global neogastropod fauna between the Paleocene and Recent and these can only be elaborated by a combination of further paleontological studies and molecular phylogenetic analysis. It should be stressed that the role of seasonality in developing polar faunas could well have been enhanced later in the Cenozoic when temperature declined significantly. This is particularly so if the production of sea ice significantly enhanced the development of diatoms and other primary producers [92]. It is also apparent that both polar regions have been subject to selective extinction events since the Early Cenozoic [93] and these, too, will have to be considered in future studies.

### Conclusions

- There is growing evidence to suggest that the origin of modern polar marine faunas can be traced back to at least the Early Cenozoic era.
- One of the largest marine clades at the present day, the Neogastropoda, exhibits not only a latitudinal gradient in species richness but also a parallel gradient in species evenness. It is likely that low evenness/high dominance is a characteristic feature of other polar marine clades too.
- The lack of evenness and preponderance of generalists in polar neogastropod families points to the key role of seasonality in primary production in structuring polar marine assemblages.
- The latitudinal gradient in seasonality may be of greater importance than the latitudinal gradient in temperature in the early evolution of polar marine faunas. Such a gradient is temperature-invariant and would have operated through the Early Cenozoic greenhouse world.
- A global analysis suggests that distinctive polar marine faunas can indeed be differentiated in the Paleocene, albeit somewhat stronger in the south than the north. The dominance of the Buccinidae, s.l. in particular at the present day may be traced back in both polar regions more than 60 m.y.
- The key role of seasonality in the evolution of polar marine assemblages may have important implications for contemporary ecosystem structure and function.

## Supporting Information

Appendix S1 (DOC)
