# Supplementary material for: Early Cenozoic Differentiation of Polar Marine Faunas
Source: PLoS One. 2013 Jan 16;8(1):e54139. doi: 10.1371/journal.pone.0054139 (PMC3546925; doi:10.1371/journal.pone.0054139)
Supplement: Appendix S1 — (DOC) [file pone.0054139.s001.doc]

**APPENDIX S1**

1. **Notes on the construction of gastropod clades**

The gastropod clades used in this study are based on the morphological analyses developed by Ponder & Lindberg [25] and Strong [94], molecular studies such as those by Harasewych et al. [95], [96] and Colgan et al. [97], [98], and the combined analyses presented by Aktipis et al. [99] and Ponder et al. [100]. Extensive use has also been made of the working classification of Gastropoda published by Bouchet & Rocroi [27]. In total nine ‘clades’ (or clade groups) have been used in this study but not all of them are true clades in the strict phylogenetic sense Estimates of their relative importance are given in percentage terms of the global modern fauna: Patellogastropoda (1%), Vetigastropoda (11%), Neritomorpha (1%), Cerithioidea (2%), Hypsogastropoda (23%), Ptenoglossa (7%), Neogastropoda (42%), Lower Heterobranchia (4%), and Opisthobranchia (9%).

**Patellogastropoda**

Generally recognised as a homogenous group, but in molecular analyses placement of the Patellogastropoda is inconsistent [99].

**Vetigastropoda**

Essentially the old Archaeogastropoda, but in molecular analyses neither the Fissurelloidea nor the Trochidae have been recovered as monophyletic. There is thus some doubt as to whether this is a truly homogenous group [99].

**Neritomorpha**

Monophyly of this group is widely recognised.

**Cerithioidea**

In a morphological analysis, Simone [101] included both the Campaniloidea and Vermetoidea within the Cerithioidea, but both molecular and sperm data suggest a more restricted definition. Ponder et al. [100] regarded the Campaniloidea as sister to the Cerithioidea + Hypsogastropoda, and placed the Vermetidae within the partially resolved ‘lower’ Hypsogastropoda.

The classification adopted by Bouchet & Rocroi [27] has been used here and includes the Ampullinidae; it is still not fully resolved and is perhaps better regarded as a grade than a clade.

**Hypsogastropoda**

A term first used by Ponder & Lindberg [25] to include the vast majority of extant caenogastropods, with the exception of the Architaenioglossa, Cerithioidea and Campaniloidea. Although the clade is fairly well defined in both morphological and molecular analyses, relationships within it remain uncertain [98], [100]. This general lack of phylogenetic resolution within the Hypsogastropoda may be due to very rapid radiation, particularly in the Early Cenozoic [100].

In this study both the Ptenoglossa and Neogastropoda , although strictly speaking Hypsogastropoda (*sensu* [100]), are treated as separate groups.

**Ptenoglossa**

Bouchet & Rocroi [27] retain Ptenoglossa as a ‘group’ name and that is the sense used here. However, in the combined analysis presented by Ponder et al. [100] ptenoglossans are scattered amongst the ‘lower’ hypsogastropods (their asiphonate group). Ptenoglossa may be a polyphyletic grouping of Eulimoidea, Janthinoidea and Triphoroidea [25], [100].

**Neogastropoda**

This large and important group is still in a state of flux but in the past it has been widely regarded as monophyletic [25], [94]. There are some inconsistencies in molecular analyses carried out to date [98], [100].

Buccinidae: The suprageneric taxonomy of buccinoidean gastropods is currently uncertain [73], [102], [103]. As presently constituted, Buccinidae s.l., a large family ranging from the tropics to both polar regions, is almost certainly paraphyletic. Southern cool-water buccinids assigned to the Subfamily (or tribe) Buccinulinae show both tropical affinities and a link to northern cool-water forms through *Lirabuccinum* [75]. It has recently been suggested that Buccinulidae at family rank should also be considered [74].

‘Turridae’: Traditionally, non-*Conus* and non-Terebridae members of the Conoidea (= Toxoglossa) have been classified within the Turridae, which has in turn been split into some nine subfamilies. However, since the pioneering anatomical synthesis of Taylor et al. [104] it has been apparent that the ‘Turridae’ is polyphyletic. Substantial resolution of this problem has now been provided by an extensive molecular phylogenetic analysis of the Conoidea which has yielded 14 principal clades [105], [106]; these have in turn been used to establish 14 families [107]. The largest and most variable taxon within the Conoidea is now the Raphitomidae, which is sister to the Mangeliidae (with the latter now including the extensive Subfamily Oenopotinae). The Borsoniidae is a rather heterogeneous group that is still not fully resolved but includes Paleocene representatives such as *Borsonia*, *Tomopleura* and *Zemacies*. Pseudomelatomidae is another large family that is also anatomically the most variable. It now includes most genera formerly placed within the Crassispirinae, and the widely occurring deep-water taxon, *Leucosyrinx*. The Turridae, s.s. is now restricted to a well-supported clade of some eight genera, including *Turris* [106], [107].

In this study all modern conoideans have been classified according to Bouchet et al. [107]; however, due to the uncertainties surrounding the precise taxonomic status of a number of Paleocene genera these have been retained within the broad heading of ‘Turridae, s.l.’ No Paleocene Conidae or Terebridae were encountered in the included faunal lists.

**Heterobranchia**

A clearly defined sister group to the Caenogastropoda which includes opisthobranchs, pulmonates and basal groups such as the Rissoelloidea, Architectonicoidea and Pyramidelloidea [99]. Caenogastropoda + Heterobranchia comprise the very well supported Apogastropoda [25], [99]. In this study the classification of Bouchet & Rocroi [27] has been adopted with an Informal Group ‘**Lower Heterobranchia**’ and an Informal Group **Opisthobranchia**. We still lack a reliable phylogeny for the Opisthobranchia [108]; it would appear to be monophyletic only when the Acteonidae is removed, and Bouchet & Rocroi [27] have included this family in the Lower Heterobranchia.

1. **Gastropod localities**

**Philippines**

Defined as approximately the state of the Philippines and combining a mixture of coral reef and non-reef habitats; faunal lists taken from Poppe [109-111] and Poppe et al. [112]. The Panglao Marine Biodiversity Project recovered ~500,000 specimens collected over a three-month period in 2004 [45]. As in New Caledonia, most of these taxa are tiny and rare, with the most diverse families being the Turridae, s.l. (Neogastropoda), Eulimidae, Triphoridae and Cerithiopsidae (all Ptenoglossa), and Pyramidellidae (Lower Heterobranchia). A considerable amount of taxonomic work remains to be done on these micromolluscs but it is likely that the total number of gastropod species in the Philippines will eventually be shown to be well in excess of 10,000 [45].

**Guam**

Coral reef and associated shallow marine habitats surrounding the largest and southernmost of the Mariana Islands. Taxonomic lists taken from [113-116]. Microgastropods <7mm poorly studied to date but opisthobranchs very well represented and studied [115]. The shallow-water marine biota of Guam is the best documented anywhere in Micronesia [116].

**New Caledonia**

Intensive sampling from coral reef and other coastal environments at three sites: Koumac, Touho, and Lifou, Loyalty Islands. Most of the species are small and rare; ~⅓ have an adult size of <4mm, and macromolluscs >40mm comprise only 8% of the fauna [117], [118]. The most speciose families, Turridae (Neogastropoda), Triphoridae, Eulimidae, Cerithiopsidae (all Ptenoglossa) and Pyramidellidae (Lower Heterobranchia) comprise ~37% of gastropod species richness. A total of approximately 3234 species obtained from <100m water depth contains a large proportion of morphospecies that have yet to be formally described. It is estimated that the true total for New Caledonia as a whole is likely to be more than twice this figure [118].

Families that are under-represented in the faunal lists at present include: Pickworthiidae, Tonnidae (Hypsogastropoda), Epitoniidae (Ptenoglossa), Haminoeidae, Philinidae, Gastropteridae, Juliidae and Placobranchidae (Opisthobranchia) [118].

**French Polynesia**

A total of 1977 species from 118 volcanic and low coral reef islands includes 491 morphospecies [119]. Some deeper-water (i.e. 200m +) taxa included and it is apparent that many micromollusc groups are still under review.

**Tropical Western Atlantic**

Species list compiled from MALACOLOG 4.1.1 [120], with the northern limit set as South Carolina (~35°N) and the southernmost on the South American coast as Guyana (5°N). All principal Caribbean islands included.

**Panamic province**

Geographic boundaries as defined in Crame [51]. Species lists taken from [121-123]; some microgastropod groups underrepresented?

**Arctic**

Species list based on all taxa occurring north of 60°N; Bering Sea and Sea of Okhotsk excluded; includes some abyssal taxa. The thirteen taxonomic sources used to compile the list are available from the author on request.

**Antarctic**

All taxa occurring south of the Polar front and recorded in the Register of Antarctic Marines Species (RAMS) [124], [125]; includes bathyal taxa.

1. **Table 1. A comparison of the sizes of the principal modern regions studied**

| **Region** | **Shelf area** | **References** |
| --- | --- | --- |
| Arctic^1^ | 5.2 x 10^6^ km^2^ | [126] |
|  |  |  |
| Philippines | 0.25 x 10^6^ km^2^ | [http://earthtrends.wri.org](http://earthtrends.wri.org/) |
| Guam | 0.0003 10^6^ km^2^ | [127] |
| New Caledonia^2^ | 0.0007 x 10^6^ km^2^ | [118] |
| French Polynesia | 0.15 x 10^6^ km^2^ | [119] |
| Tropical Western Atlantic | 0.8 x 10^6^ km^2^ | http://earthtrends.wri.org |
| Panamic province^3^ | 0.25 x 10^6^ km^2^ | [127], [128] |
|  |  |  |
| Antarctic^1^ | 4.6 x 10^6^ km^2^ | [129] |

**Notes:**

^1^ Includes both ice-covered and ice-free shelf

^2^ Covers three sites: Koumac, Touho and Lifou

^3^ Includes Galapagos Islands

It should be noted that the practice of dividing total biome species richness by biome area to derive an estimate of species per unit area must be treated with some caution. This is because the species-area relationship is essentially non-linear [130].

1. **Table 2. Taxonomic diversity statistics for the Neogastropoda**

|  | **No. families/clade** | **No. species/family** |
| --- | --- | --- |
| 1. Modern fauna |  |  |
| Arctic - Neogastropoda | 9 | 20.11 |
| Tropics – Neogastropoda (median value) | 19.00 | 48.32 |
| Antarctic - Neogastropoda | 9 | 16.22 |
|  |  |  |
| 1. Paleocene fauna |  |  |
| West Greenland | 14 | 6.21 |
| Tropics – NW Europe | 17 | 7.59 |
| Southern high lats | 10 | 8.60 |

1. **Paleocene localities and faunal lists**
2. West Greenland (63°N)

Taxonomic list taken from [65], [66], with revisions from the following sources: [131-134]; Paleobiology Database (=PBDB), May 2012.

The fossils are from the Sonja and Turritellakloft members of the Agatdal Formation; Middle Paleocene (Selandian) age. Fauna has both warm- and cold-water affinities and shows similarities with Danian faunas from both Denmark and North and South Dakota [65]. Many of the identifications are still only provisional but small taxa (i.e. <10mm) are well represented.

1. Copenhagen, Denmark (49°N)

Fauna from the Middle Paleocene (Selandian) Lelinge Greensand taken from [135], [136] , with revisions from: [43], [137] and PBDB, May 2012. This fauna differs from that of the older (Danian) fauna of Fakse and has more temperate affinities. It does include a number of tiny specimens.

1. Fakse, Denmark (48.5°N)

Predominantly a fauna of small (<10mm) to tiny (<5mm) forms sieved from a bioclastic limestone; epifaunal and hard substrate-associated taxa predominate. Species list taken from [138], with revisions from [139], [140]; Middle Danian in age. The coral limestones of Fakse comprise a series of coral – bryozoan bioherms but no true reefs [141]. Molluscan fauna has distinct tropical/subtropical affinities.

1. Mons, Belgium (44°N)

A prolific fauna from the bioclastic limestones comprising the Calcaire grossier de Mons. Faunal list taken from [142], with revisions from [132], [143] and PBDB, May 2012. Cerithioidea particularly well represented and small taxa abundant. Danian in age, and probably mid- to late Danian.

1. Vigny (Paris Basin), France (43°N)

Abundant but on the whole poorly preserved fossils from the Danian Calcaire de Vigny; many casts and moulds of aragonitic taxa but no shell material. This fauna is still incompletely known, but one reliable estimate puts the total number of gastropod species at no less than 170 [132]; both hypsogastropod and neogastropod lists incomplete. List of taxa taken from: [132], [144-147]; PBDB, May 2012. Perireefal depositional environment [41].

1. Southern Poland (40°N)

Faunal list from the Babica Clays, Middle Carpathians; preservation very variable; Late Paleocene (Thanetian) age. Data taken from Krach [148], with amendments from [132] and the PBDB, May 2012.

1. Gulf Coast, Aquia Formation (39°N)

The Aquia Formation comprises Early Paleocene (Danian) shales and highly glauconitic sandstones from Virginia and Maryland; fossils occur in locally indurated shell beds. Faunal list taken from [149], [150], with revisions from [151] and the PBDB, May 2012.

1. Salzburg, Austria (38°N)

Fauna obtained from a sequence of dark, sandy marls with intercalated coarse, glauconitic sandstones comprising the Oiching Formation of the Haunsberg region north of Salzburg; predominantly Danian in age, although in places may range into mid- or even late Paleocene. Faunal data taken from [152-157]; some tiny forms, such as rissoids and liotiids, well represented.

1. Gulf Coast, Kincaid Formation (36°N)

A comparatively thin lithostratigraphic unit (>9m) in Texas of richly fossiliferous limestones grading laterally into shales. Basal Danian in age; amongst one of the most complete earliest Paleocene sequences anywhere in the world. Faunal list taken from: [149], [150], [158], [159]; PBDB, May 2012.

1. Gulf Coast, Porters Creek Formation (35°N)

A sequence of silty clays, sandy marls and glauconitic sandstones comprise this uppermost formation within the Midway Group in Mississippi and western Alabama; Selandian in age. Faunal list taken from: [44], [149], [150], [160].

1. Gulf Coast, Wills Point Formation (35°N)

The Wills Point Formation comprises a fossiliferous sequence of clays, silty clays and sandy marls with subordinate limestones exposed in Texas; Danian – Selandian. Faunal list taken from [150], with revisions from [44], [161] and PBDB, May 2012.

1. Gulf Coast, Clayton Formation (35°N)

A sequence of sandstones, sandy limestones, limestones, minor clays and mudstones exposed in Mississippi, Georgia, Alabama and southern Illinois; Danian in age. Faunal list taken from: [150], [160], [162].

1. Gulf Coast, Naheola Formation (35°N)

A sequence of silty clays, sandy marls and glauconitic sandstones exposed in Mississippi and western Alabama; Selandian in age. Faunal list taken from: [149], [150], [160].

1. S.W. Nigeria (2°S)

The Ewekoro Formation comprises a thin (>17m) sequence of richly fossiliferous coquinoidal limestones but no coral reefs; Danian – Selandian in age. Faunal list taken from: [163], [164] and PBDB, May 2012. The gastropod fauna is dominated by small taxa (<1cm) and shows similarities with both Mokattam, Egypt and especially the Upper Ranikot Formation of S.E. Pakistan. Adegoke [163] indicates that there are still many undescribed taxa within this fauna.

o) S.E. Pakistan (5°S)

The Upper Ranikot Formation of western Sind, S.E. Pakistan comprises a thick (>240m) sequence of fossiliferous limestones interbedded with sandstones, shales and clays; Thanetian in age. Colonial corals are present within the limestones but there is no evidence of reefs. Faunal list taken from: [165-167]. Fauna in need of full revision; Vredenburg [167] indicates that a number of new taxa have not yet been formally described, and the prolific Turritellidae are under-studied.

N.B. A Danian fauna from the informal “Cardita beaumonti beds” of Sind [168], [169] is very incomplete. It is uncertain whether it correlates directly with the “Cardita beaumonti beds” identified in Beluchistan, western Pakistan [170]. There is some evidence to suggest that the latter is from a lower stratigraphic level and brackish/lagoonal in aspect. A further incomplete Danian fauna is known from the Samana Range, N.W. Frontier Province [171].

1. Southern Patagonia (55°S)

Faunal list restricted to a series of localities in southernmost Patagonia attributable to either the topmost Cerro Cazador Formation or the overlying Cerro Dorotea Formation [172-174]; PBDB, May 2012). A Danian fauna showing strong affinities to Zinsmeister’s [34] Weddellian Province [173].

1. Southern New Zealand (56°S)

Essentially the Danian Wangaloa Formation of the southern South Island and its stratigraphical equivalents: Broken River Formation; Kauru Formation; Abbotsford Formation [35], [36]. In compiling this list use was also made of collections stored at GNS Science, Lower Hutt, including the extensive A. Grebneff Collection; PBDB (May 2012) also used. Micromolluscs included.

1. S.E. Australia (61°S)

The Pebble Point Formation of the Otway Basin, Victoria comprises >60m of essentially fine- to coarse-grained sandstone lithologies; Selandian – Thanetian in age. Faunal list compiled from: [36], [175], [176]; PBDB, May 2012. Fauna contains several characteristic Weddellian Province elements.

1. Antarctic Peninsula (64°S)

A Danian fauna comprising the topmost “Unit 10” of the López de Bertodano Formation and the overlying Sobral Formation. The latter comprises some 300m of siliciclastic lithologies ranging from sandy mudstones to matrix-rich conglomerates. Faunal list compiled from [19], [67], [177]; use was also made of the W.J. Zinsmeister Collection, Paleontological Research Institution, Ithaca, N.Y., and the B.A.S. collections.

**Appendix References**

(References cited in the main paper are not repeated here)

1. Strong EE (2003) Refining molluscan characters: morphology, character coding and a phylogeny of the Caenogastropoda. Zool J Linn Soc, 137, 447-554.
2. Harasewych MG, Adamkewicz SL, Blake JA, Saudek D, Spriggs T, et al. (1997) Neogastropod phylogeny: a molecular perspective. J Moll Stud, 63, 327-351.
3. Harasewych MG, Adamkewicz SL, Plassmeyer M, Gillevet PM (1998) Phylogenetic relationships of the lower Caenogastropoda (Mollusca, Gastropoda, Architaenioglossa, Campaniloidea, Cerithioidea) as determined by partial 18S sequences. Zool Scripta, 27, 361-372.
4. Colgan DJ, Ponder WF, Eggler,PE (2000) Gastropod evolutionary rates and phylogenetic relationships assessed using partial 28S rDNA and histone H3 sequences. Zool Scripta ,29, 29-63.
5. Colgan DJ, Ponder WF, Beacham E, Macaranas JM (2007) Molecular phylogenetics of Caenogastropoda (Gastropoda: Mollusca). Mol Phylogen Evol, 42, 717-737.
6. Aktipis SW, Giribet G, Lindberg DR, Ponder WF (2008) Gastropoda. An overview and analysis. Phylogeny and evolution of the Mollusca (ed. W.F. Ponder, D.R. Lindberg), pp. 209-237. Univ California Press, London.
7. Ponder WF, Colgan DJ, Healy JM, Nutzel A, SimoneLRL, et al. (2008) Caenogstropoda. Phylogeny and evolution of the Mollusca (ed. W.F. Ponder, D.R. Lindberg), 331-383. Univ. California Press, London.
8. Simone LR (2001) Phylogenetic analyses of Cerithioidea (Mollusca, Caenogastropoda) based on comparative morphology. Arquiv Zool Sã0 Paulo, 36*,* 147-263.
9. Haasl DM (2000) Phylogenetic relationships among nassariid gastropods. J Paleont, 74, 839-852.
10. Amano K, Vermeij GJ (2003) Evolutionary adaptation and geographic spread of the Cenozoic buccinid genus *Lirabuccinum* in the North Pacific. J Paleont, 77, 863-872.
11. Taylor JD, Kantor YI, Sysoev AV (1993) Foregut anatomy, feeding mechanisms and classification of the Conoidea. Bull Nat Hist Mus Lond (Zool), 59, 125-170.
12. Puillandre N, Samadi S, Boisselier MC, Sysoev AV, Kantor YI et al. (2008) Starting to unravel the toxoglossan knot: molecular phylogeny of the ‘turrids’ Neogastropoda: Conoidea). Mol Phylog Evol ,47, 1122-1134.
13. Puillandre N, Kantor YI, Sysoev A, Couloux A, Meyer C et al. (2011) The dragon tamed? A molecular phylogeny of the Conoidea (Gastropoda). J Moll Stud, 77, 259-272.
14. Bouchet P, Kantor YI, Sysoev A, Puillandre N (2011) A new operational classification of the Conoidea (Gastropoda). J Moll Stud, 77, 273-308.
15. Wägele H (2008) Heterobranchia I. The Opisthobranchia. Phylogeny and evolution of the Mollusca (ed. W.F. Ponder, D.R. Lindberg), 385-408. Univ California Press, London.
16. Poppe GT (ed.) (2008a) Philippine Marine Mollusks. Vol. 1, Gastropoda Part 1. Conch Books, Hackenheim, 758p.
17. Poppe GT (ed.) (2008b) Philippine Marine Mollusks, Vol. 2, Gastropoda Part 2. Conch Books, Hackenheim, 848p.
18. Poppe GT (ed.) (2010) Philippine Marine Mollusks, Vol. 3, Gastropoda Part 3 & Bivalvia Part 1. Conch Books, Hackenheim, 665p.
19. Poppe GT, Tagaro SP, Salisbury R (2009) New species of Mitridae and Costellariidae from the Philippines with additional information on the Philippine species in these families. Visaya, Suppl. 4, 1-87.
20. Kay EA (1995) Pacific island marine molluscs: systematics. Marine and coastal biodiversity in the tropical island Pacific region Vol. 1. Species, systematics and information management priorities (ed. J.E. Maragos, M.N.A. Peterson, L.G. Eldredge, J.E. Bardach , H.F. Takeuchi), 135-159. East-West Center, Honolulu.
21. Smith BD (2003) Prosobranch gastropods of Guam. Micronesica**,** 35-36, 244-270.
22. Carlson C, Hoff PJ (2003) The opisthobranchs of the Mariana Islands. Micronesica, 35-36, 271-293.
23. Paulay G (2003) Marine diversity of Guam and the Marianas: overview. Micronesica, 35-36, 3-25.
24. Bouchet P, Lozouet P, Maestrati, P, Heros V (2002) Assessing the magnitude of species richness in tropical marine environments: exceptionally high numbers of molluscs at a New Caledonia site. Biol J Linn Soc, 75, 421-436.
25. Héros V, Lozouet P, Maestrati P, von Cosel R, Brabant D, et al. (2007) Compendium of marine species of New Caledonia (ed. C.E. Payri, B. Richer de Forges), 199-254, Doc. Sci. Tech*.* 117.
26. Trondlé J, Boutet M (2009) Inventory of marine molluscs of French Polynesia. Atoll Res Bull, 570, 1-87.
27. Rosenberg G (2009) Malacolog 4.1.1: A database of Western Atlantic marine Mollusca. [WWW database (version 4.1.1)]. URL http:// www. malacolog.org
28. KeenAM (1971) Sea shells of tropical west America 2^nd^ edn. Stanford Univ Press, Stanford, CA., 1064p.
29. Skoglund C (1992) Additions to the Panamic Province gastropod (Mollusca) literature 1971 to 1992. The Festivus, 24, Suppl, 169p.
30. Skoglund C (2002) Panamic Province molluscan literature. Additions and changes from 1971 through 2001. The Festivus, 33, Suppl, 286p.
31. De Broyer C, Danis B (ed.) (2010) SCAR MarBIN: The Antarctic Marine Biodiversity Information Network. www electronic publication. Available online at the SCAR MarBIN website

<http://www.scarmarbin.be/>

1. Griffiths HJ (2010) Antarctic marine biodiversity – what do we know about the distribution of life in the Southern Ocean? PloS ONE, **5**, e11683.
2. Jakobsson M (2002) Hypsometry and volume of the Arctic Ocean and its constituent seas. Geochem Geophys Geosyst, 3, doi:10.1029/2001GC000302.
3. Spalding MD, Ravilious C, Green EP (2001) World atlas of coral reefs. Univ California Press, Berkeley, CA, 424p.
4. Roy K, Jablonski D, Valentine JW, Rosenberg G (1998) Marine latitudinal diversity gradients: tests of causal hypotheses. Proc Nat Acad Sci USA, 95, 3699-3702.
5. Clarke A, Johnston NM (2003) Antarctic marine benthic diversity. Oceanog Mar Biol Ann Rev, 41, 47-114.
6. Rosenzweig ML (1995) Species diversity in space and time. Cambridge Univ. Press, Cambridge, UK, 436p.
7. Pacaud, J-M, Schnetler KI (1999) Revision of the gastropod family Pseudolividae from the Paleocene of West Greenland and Denmark. Bull Geol Soc Denmark , 46, 53-67.
8. Pacaud J-M (2004) Révision des mollusques du Danien (Paléocène inférieur) du Bassin de Paris. 1. Gastropoda: Patellogastropoda et Vetigastropoda (*pro parte*). Geodiversitas, 26, 577-629.
9. Merle D, Pacaud J-M (2003) New species of *Eocithara* Fischer, 1883 (Mollusca, Gastropoda, Harpidae) from the Early Paleogene with phylogenetic analysis of the Harpidae. Geodiversitas, 26, 61-87.
10. Schnetler KI, Petit RE (2010) Revision of the gastropod family Cancellariidae from the Paleocene of Nuussuaq, West Greenland. Cainozoic Res, 7, 3-26.
11. Ravn JPJ (1939) Études sur les mollusques du Paléocène de Copenhague. Biol Skr, 1, 106p.
12. Schnetler KI (2001) The Selandian (Paleocene) mollusc fauna from Copenhagen, Denmark: the Poul Harder 1920 collection. Bull Geol Surv Denmark, 37, 85p.
13. Collins JSH (1995) A new crab, *Rogeus robustis*, from the Middle Paleocene of Denmark. Bull Mizunami Fossil Museum, 22, 61-65.
14. Ravn JPJ (1933) Études sur les pélécypodes et gastropods daniens du Calcaire de Faxe. K danske Vidensk Selsk Skr, 5, 72p.
15. Schnetler KI, Lozouet P, Pacaud J-M (2001) Revision of the gastropod family Scissurellidae from the Middle Danian (Paleocene) of Denmark. Bull Geol Soc Denmark, 48, 79-90.
16. Schnetler KI, Petit RE (2006) Revision of the gastropod family Cancellariidae from the Danian (Early Paleocene) of Fakse, Denmark. Cainozoic Res, 4, 97-108.
17. Bernecker M, Weidlich O (1990) The Danian (Paleocene) Coral Limestone of Fakse, Denmark: A model for ancient aphotic, azooxanthellate coral mounds. Facies, 22, 103-138.
18. Glibert M (1973) Revision des Gastropoda du Danien et du Montien de la Belgique. I Les Gastropoda du Calcaire de Mons. Mem Inst Royal Sci Nat de Belgique, 173, 116p.
19. Pacaud J-M (1998) Nouvelles données sur le genre *Popenoeum* (Mollusca, Pseudolividae), remarques taxinomiques sue une espèce ubiquiste du Paléocène Inférieur, *Popenoeum ambiguum* (Binkhorst, 1861: *Pyrula*) nov comb. Cossmanniana, 5, 1-28.
20. Pacaud J-M (2007) Nouveautés nomenclaturales et taxonomiques introduites par Alcide d’Orbigny dans le *Prodrome* (1850, 1852) pour les espèces du Paléocène et de l’Éocène. Geodiversitas, 29, 17-85.
21. Pacaud J-M (2009) Révision des mollusques du Danien (Paléocène inférieur) du Bassin de Paris. 2. Gastropoda: Neritomorpha. Revue de Paléobiologie, Genève, 28, 349-369.
22. Pacaud J-M, Merle D (2002) Alcide d’Orbigny, un précurseur dans l’étude de la faune du Danien du basin de Paris. CR Acad Sci Palevol ,1, 587-598.
23. Pacaud J-M, Merle D, Meyer J-C (2000) La faune danienne de Vigny (Val-d’Oise, France): importance pour l’étude de la diversification des mollusques au début du Tertiaire. CR Acad Sci Paris Sci de la Terre et des plan, 330, 867-873.
24. Krach W (1963) Mollusca of the Babica Clays (Paleocene) of the Middle Carpathians Pt1 Gastropoda. Stud Geol Polon. 14, 151p.
25. Palmer KVW, Brann DC (1965) Catalogue of the Paleocene and Eocene Mollusca of the southern and eastern Unites States Part I. Pelecypoda, Amphineura, Pteropoda, Scaphopoda, and Cephalopoda. Bull Am Paleont, 48 (218), 466p.
26. Palmer KVW, Brann DC (1966) Catalogue of the Paleocene and Eocene Mollusca of the southern and eastern Unites States Part II. Gastropoda. Bull Am Paleont ,48 (218), 1057p.
27. Ward LW, Waller TW (1988) A new species of *Pulvinites* (Mollusca: Bivalvia) from the Upper Paleocene Paspotansa Member of the Aquia Formation in Virginia. J Paleont ,62, 51-55.
28. Traub F (1979) Weitere Paleozän-Gastropoden aus dem Helvetikum des Haunsberges nördlich von Salzburg. Mitt Bayer Staats Paläont hist Geol ,19, 93-123
29. Traub F (1980) Weitere Paleozän-Gastropoden aus dem Helvetikum des Haunsberges nördlich von Salzburg. 1 Fortsetzung. Mitt Bayer Staats Paläont hist Geol, 20, 29-49.
30. Traub F (1981) Weitere Paleozän-Gastropoden aus dem Helvetikum des Haunsberges nördlich von Salzburg. 2 Fortsetzung. Mitt Bayer Staats Paläont hist Geol, 21, 41-63.
31. Traub F (1984) Weitere Paleozän-Gastropoden aus dem Helvetikum des Haunsberges nördlich von Salzburg. 3 Fortsetzung. Mitt Bayer Staats Paläont hist Geol, 24, 3-26.
32. Traub F (1989) Weitere Paleozän-Gastropoden aus dem Helvetikum des Haunsberges nördlich von Salzburg. 4 Fortsetzung. Mitt Bayer Staats Paläont hist Geol, 29, 85-108.
33. Traub F, Werner W (1993) Biostratigraphie Einstufung der Gastropoden aus dem Paleozän (Tertiär) des Haunsberges (N Salzburg, Österreich) anhand der internationalen Plankton-Foraminiferen-Zonierung. Zitteliana, 20, 369-378.
34. Hansen TA (1993a) Early Tertiary radiation of marine molluscs and the long-term effects of the Cretaceous – Tertiary extinction. Paleobiology, 14, 37-51.
35. Hansen TA (1993b) Patterns of molluscan extinction and recovery across the Cretaceous – Tertiary boundary in east Texas; report on new outcrops. Cret Res, 14, 685-706.
36. Toulmin LD (1977) Stratigraphic distribution of Paleocene and Eocene fossils in the Eastern Gulf Coast region. Geol Surv Alabama Monograph, 13, 602p.
37. Beu AG, Maxwell PA (1987) A revision of the fossil and living gastropods related to *Plesiotriton* Fischer, 1884 (Family Cancellariidae, Subfamily Plesiotritoninae n. subfam. NZ Geol Surv Pal Bull 54, 71p.
38. Cope KH, Utgaard JE, Master JM, Feldmann RM (2005) The fauna of the Clayton Formation (Paleocene, Danian) of southern Illinois: a case of K/P survivorship and Danian recovery. Bull Mizunami Fossil Museum, 32, 97-108.
39. Adegoke OS (1973) Paleocene molluscs from Ewekoro, southern Nigeria. Malacologia, 14, 19-27.
40. Adegoke OS (1977) Stratigraphy and paleontology of the Ewekoro Formation (Paleocene) of southwestern Nigeria. Bull Am Paleont, 71, 379p.
41. Cossmann M, Pissarro G (1926) The Mollusca of the Ranikot Series Part I Cephalopoda and Gastropoda. Mem Geol Surv India Pal Indica, 3, 1-83.
42. Vredenburg EW (1926) Introductory note on the stratigraphy of the Ranikot Series. Mem Geol Surv India Pal Indica, 3, v-xix.
43. Vredenburg EW (1928) A supplement to the Mollusca of the Ranikot Series. Mem Geol Surv India Pal Indica, 10, 75p.
44. Cossmann M, Pissarro G (1927) The Mollusca of the Ranikot Series Part II Brachiopoda and Lamellibranchiata (together with some species from the *Cardita beaumonti* beds). Mem Geol Surv India Pal Indica, 10, 31p.
45. Douvillé H (1929) Les couches ā *Cardita beaumonti*: Fascicule II. Les couches ā *Cardita beaumonti* dans le Sind. Mem Geol Surv India Pal Indica, 10, 13p.
46. Douvillé H (1928) Les couches ā *Cardita beaumonti*. Mem Geol Surv India Pal Indica, 10, 25p.
47. Cox LR (1930) The fossil fauna of the Samana Range and some neighbouring areas. Part VIII. The Mollusca of the Hangu Shales. Mem Geol Surv India Pal Indica, 15, 129-222.
48. Hünicken MA (1955) Depósitos Neocretácicos y Terciarios del extreme SSW de Santa Cruz (Cuenca Carbonifera de Rio Turbio). Revta Inst nac Invest Cienc nat Cienc geol, 4, 1-161.
49. Griffin M, Hünicken MA (1994) Late Cretaceous – Early Tertiary gastropods from southwestern Patagonia, Argentina. J Paleont, 68, 257-274.
50. del Rio CJ (2002) Moluscos del Terciario marino. GeologÍa y Recursos Naturales de Santa Cruz (ed. M.J. Haller), II-9, 1-22.
51. Darragh TA (1994) Paleocene bivalves from the Pebble Point Formation, Victoria, Australia. Proc R Soc Vic, 106, 71-103.
52. Darragh TA (1997) Gastropoda, Scaphopoda, Cephalopoda and new Bivalvia of the Paleocene Pebble Point Formation, Victoria, Australia. Proc R Soc Vic, 109, 57-108.
53. Harasewych MG, Oleinik A, Zinsmeister WJ (2009) The Cretaceous and Paleocene pleurotomariid (Gastropoda: Vetigastropoda) fauna of Seymour Island, Antarctica. J. Paleont, 83, 750-766.
